# Micro-hardness of root canal dentin using various irrigating solutions: An *in vitro* study

**DOI:** 10.6026/973206300211057

**Published:** 2025-05-31

**Authors:** Richa Pathak, Meetu Mathur, Diganta Kumar Borah, Amit Kumar, Sonam Agrawal, Chinmayee Prusty, Pratik Surana

**Affiliations:** 1 Department of Conservative Dentistry and Endodontics, Buddha Institute of Dental Sciences and Hospital, Patna, Bihar, India; 2 Department of Conservative Dentistry and Endodontics, Rajasthan Dental College and Hospital, Nirwan University, Jaipur, Rajasthan, India; 3 Department of Conservative Dentistry and Endodontics, Regional Dental College, Guwahati, Assam, India; 4 Department of Dentistry, Lord Buddha Koshi Medical College and Hospital, Saharsa, Bihar, India; 5 Department of Oral Pathology and Microbiology, Maitri College of Dentistry and Research Centre, Durg, Chhattisgarh, India; 6 Department of Pedodontics and Preventive Dentistry, Kalinga Institute of Dental Sciences, Kalinga Institute of Industrial Technology (KIIT) Deemed to be University, Bhubaneswar, Odisha, India; 7 Department of Pedodontics and Preventive Dentistry, Maitri College of Dentistry and Research Centre, Durg, Chhattisgarh, India

**Keywords:** Micro-hardness, irrigating solution, root canal dentin, vickers micro-hardness tester (VMT)

## Abstract

Evaluation of changes in dentin microhardness during root canal irrigation using eighty extracted mandibular premolars is of interest. After decoronation and halving, baseline microhardness was measured with a Vickers tester. The samples were divided into four groups: control (Normal Saline), 3% Sodium Hypochlorite, 17% EDTA, and 2% Chlorhexidine Gluconate, with each immersed in their respective irrigants for 15 minutes at 37°C. Results showed decreased microhardness in all groups except the control, with 17% EDTA causing the most significant reduction. Notable differences were found between groups 2, 4, and 1, but not between 1 and 3. Consequently, 2% Chlorhexidine is considered a suitable irrigation solution as it did not negatively impact dentin microhardness.

## Background:

Removing microorganisms is crucial in endodontic treatment. Relying solely on mechanical instrumentations of the infected canal may not be enough to achieve this goal. Thus, using irrigating solutions during root canal irrigation is a vital aspect of the endodontic process, as it aids in cleaning and disinfecting areas that may not be adequately addressed by the instruments used [1, 2]. During this irrigation, these endodontic solutions interact closely with the dentin surface and can affect its chemical composition and physical properties. Therefore, understanding how the irrigation solution impacts radicular dentin is essential. Variations in the calcium-phosphorus ratio can alter the initial balance of organic and inorganic components in dentin, affecting its microhardness, solubility, permeability, and surface roughness [3]. Commonly utilized agents for endodontic irrigation include citric acid, hydrogen peroxide (H2O2), ethylenediaminetetraacetic acid (EDTA), sodium hypochlorite (NaOCl) and chlorhexidine (CHX) [4, 5- 6]. These agents provide a range of advantages, including antimicrobial properties, the removal of the smear layer, and the dissolution of organic tissues [7]. Notably, NaOCl, CHX, and EDTA are favored for their effectiveness in breaking down organic material, clearing the smear layer, and offering strong antimicrobial action [8]. However, it's important to note that these solutions can impact the chemical composition of dentin, especially concerning the calcium levels in hydroxyapatite crystals, which can affect crucial dental properties like microhardness [4]. By assessing dentin microhardness, we can draw conclusions about changes in the physical and chemical characteristics of dentin, including its mineral content and elasticity modulus [9, 10]. A decrease in dentin microhardness results in a corresponding decline in the modulus of elasticity of the dentin. Therefore, it is of interest to compare the decrease in dentin microhardness, a study was carried out using several irrigating solutions, including normal saline, 17% EDTA, 3% NaOCl, and 2% CHX.

## Materials and Methods:

The current in-vitro study was carried out in eighty orthodontically extracted mandibular premolars that were caries-free, unharmed root apices, fracture-free, no restoration; single rooted was included in the study. Caries, fracture, restoration, and multi-rooted teeth were excluded. Collected tooth were disinfected with Chloramine-T (0.5%) solution for 2 days and cleaned by using an ultrasonic scaler to get rid of both soft and hard tissue debris and kept in thymol (0.1%) solution. Diamond impregnated disc was utilized to de-coronate the samples at a level of CEJ to uniform the canal length.

Endodontic procedure was not carried. By making buccal and lingual grooves on the outside of the roots without entering the canals, a longitudinal section was created by double-faced diamond disc that was cooled in water. The roots were then split in half using a chisel. The sectioned specimens were examined for cracks under a stereomicroscope. Thus, eighty samples were enrolled and carborandum-disc with water coolant utilized for grounding and polishing with buff consuming aluminium oxide powder with distilled water. Even blocks were prepared of diameter of 2x2cm with polished side facing outwards using plastic rings utilizing self-cure acrylic resin and after setting rings were removed. Re-polishing was done to clean the polished surface of tooth. MH1 (baseline microhardness) was recorded for all samples utilizing VMT tailored with a 200g weight. The Vickers hardness number was calculated at the apical-third of root canal dentin after the diamond indenter was left to penetrate for 20 seconds at an average distance of 100µm for 3 times and an average measurement was obtained to get the representative hardness value for each specimen. The samples were categorized into 4 groups at random (n=20) and dipped in a plastic jar comprising irrigating solutions at 37ºC for 15 min. All the samples were rinsed with distilled water and patted dry. MH2 (microhardness after immersion) was recorded for each sample. One-way ANOVA and Tukey post hoc test were utilized to gather and statistically analyze the data with p≤0.05.

Group 1: Normal saline

Group 2: 3% NaOCl

Group 3: 17% EDTA solution

Group 4: 2% CHX

## Results:

Table 1 delineates mean micro-hardness comparison among the experimental groups showing that except for the control group, every evaluated specimen shown a drop in microhardness values after applying various irrigating solutions. 17% EDTA significantly decrease in MH2 followed by group 2, 4, 1. Except for groups 1 and 3, intergroup comparison yields statistically significant findings.

## Discussion:

The long-term success of root canal treatment largely hinges on the effectiveness of instrumentation, irrigation, disinfection, and ultimately, the filling of the root canal system. When irrigating, both the coronal and radicular dentin are exposed to the irrigating solutions, which can affect the physical and chemical properties of the dentin, including its hardness. Consequently, this study aimed to assess the impact of various endodontic irrigating solutions such as normal saline, 17% EDTA, 3% NaOCl, and 2% CHX [11]. Micro-hardness testing is a common approach to examine subtle changes in hardness, whether deliberate or not, and is recognized for being simple and non-destructive. The Vickers hardness test, which is appropriate for measuring the micro-hardness of deeper dentin, was employed due to its effectiveness in evaluating changes in the surface of deeper hard tissue structures [11, 12]. Single-rooted lower premolars featuring straight, single canals were chosen, as they are the most frequently utilized extracted teeth for orthodontic correction [13]. The results provided valuable insights into the effects of various endodontic irrigants on the micro-hardness of root canal dentin. A decrease in the microhardness values of the dentin was observed in all experimental groups following the application of the irrigants, which raises several important considerations concerning the implications for clinical practice and future research. The use of endodontic irrigants is essential for effective root canal treatment, particularly for the removal of debris, biofilm disruption, and disinfection of the canal system [14]. However, the findings indicate that these irrigants, especially 3%NaOCl and 17% EDTA, can negatively affect the mechanical properties of dentin. The reduction in microhardness suggests that these substances may lead to structural changes in the dentin matrix [8]. 17% EDTA is utilized primarily for its chelating properties, which help in the removal of the inorganic component of dentin. It was notable that specimens treated with 17%EDTA experienced reduced micro-hardness. This demonstrates its efficacy in decalcifying dentin, which compromises the dentin's structural strength, an outcome provoking a cautionary approach to its clinical application, especially in cases where dentin integrity is paramount [15]. NaOCl is known for its potent antimicrobial properties and its ability to dissolve organic tissue. The study found that dentin exposed to NaOCl demonstrated a significant decrease in microhardness. This can be attributed to the chemical interaction between NaOCl and the organic components of dentin, leading to reduction in the mineral content and a subsequent loss of mechanical integrity [16]. In contrast, 2% CHX known for its antibacterial properties, also resulted in decreased microhardness, but to a lesser extent compared to NaOCl and EDTA. This suggests that while CHX may be less detrimental to dentin compared to other agents, clinicians should still be cognizant of potential adverse effects when it comes to maintaining dentin integrity. The decrease in dentin microhardness post-irrigation has significant clinical implications. Compromised dentin strength may lead to increased susceptibility to fractures, particularly in teeth subjected to occlusal forces. In root canal-treated teeth, maintaining the integrity of the remaining structure is critical for long-term success. Clinicians must strike a balance between effective disinfection and preservation of dentin hardness. Therefore, integrating the use of less erosive solutions or adjusting concentrations and exposure time may mitigate detrimental effects [12].

Agarwal *et al.* (2024) carried out a comprehensive review focusing on how various irrigating solutions, along with their combinations and modes of activation, affect the microhardness of root canal dentin. The relationship between different irrigants and dentin microhardness is intricate and shaped by factors such as their concentration, the length of contact, and their chemical characteristics. The significant differences in hardness values reported in the studies stem from variables like specimen preparation, errors in diagonal length measurements, differences in chemical makeup, the age of the teeth, and their anatomical locations. The review indicates that both the concentration and the contact duration of NaOCl and EDTA can substantially diminish the microhardness of dentin's organic and inorganic components [4]. In a similar study by Massoud *et al.* the microhardness of root canal dentin was compared after using different irrigants. These included 2.5% Sodium Hypochlorite, 17% ethylene diamine tetra-acetic acid (EDTA) followed by 10 ml of 2.5% NaOCl, 10 ml of 2.5% NaOCl followed by 10 ml of 2% chlorhexidine digluconate, and 10 ml of 2.5% NaOCl followed by 10 ml of distilled water, then 10 ml of 2% CHX. All the irrigating solutions utilized in this study significantly reduced dentin microhardness. The combination of 17% EDTA followed by 2.5% NaOCl resulted in the greatest percentage decrease in microhardness values [17]. The study limits the complex *in-vivo* biological environment such as dentin permeability, restorative materials interaction, and pulp's physiological responses can alter outcomes. Future studies need to evaluate the effects of varying concentrations, exposure times, and combinations of irrigants in a more clinically relevant model, use of protective agents or treatments post-irrigation providing insight into optimizing irrigation protocols without compromising dentin integrity.

## Conclusion:

The critical need to understand the effects of different endodontic irrigating solutions on the microhardness of root canal dentin is reinforced. Since 2%CHX has no influence on the microhardness of root canal dentin, it appears to be a suitable irrigation solution.

## Figures and Tables

**Table 1:** Comparison of mean micro- hardness among the four groups

**Groups**	**Mean ± SD of MH1**	**Mean ± SD of MH2**	**Intergroup comparison (MH2)**	
Group 1	56.65±1.54	56.01±1.34	Group	p-value
Group 2	56.25±1.23	53.62±1.78	2-Jan	0.001*
Group 3	57.81±1.45	40.98±1.99	3-Jan	0.0001*
Group 4	56.22±1.61	55.23±1.34	4-Jan	0.01
F value	4.34	102.85	3-Feb	0.001*
p-value	0.6	0.001	4-Feb	0.001*
			4-Mar	0.0001*
SD-Standard Deviation,
* Statistically Significant
